# Visual detection of H3 subtype avian influenza viruses by reverse transcription loop-mediated isothermal amplification assay

**DOI:** 10.1186/1743-422X-8-337

**Published:** 2011-07-05

**Authors:** Yi Peng, Zhixun Xie, Jiabo Liu, Yaoshan Pang, Xianwen Deng, Zhiqin Xie, Liji Xie, Qing Fan, Jiaxun Feng, Mazhar I Khan

**Affiliations:** 1 Department of Biotechnology, Guangxi Veterinary Research Institute, 51 You Ai Road, Nanning, Guangxi 530001, China; 2 Department of Pathobiology and Veterinary Science, University of Connecticut, 61 North Eagleville Road Storrs, CT 06269-3089, USA; 3 Guangxi Key Laboratory of Subtropical Bioresources Conservation and Utilization, College of Life Science and Technology, Guangxi University, 100 Daxue Road, Nanning, Guangxi 530004, China

**Keywords:** Loop-mediated isothermal amplification, H3 subtype, avian influenza

## Abstract

**Background:**

Recent epidemiological investigation of different HA subtypes of avian influenza viruses (AIVs) shows that the H3 subtype is the most predominant among low pathogenic AIVs (LPAIVs), and the seasonal variations in isolation of H3 subtype AIVs are consistent with that of human H3 subtype influenza viruses. Consequently, the development of a rapid, simple, sensitive detection method for H3 subtype AIVs is required. The loop-mediated isothermal amplification (LAMP) assay is a simple, rapid, sensitive and cost-effective nucleic acid amplification method that does not require any specialized equipment.

**Results:**

A reverse transcription loop-mediated isothermal amplification (RT-LAMP) assay was developed to detect the H3 subtype AIVs visually. Specific primer sets target the sequences of the hemagglutinin (HA) gene of H3 subtype AIVs were designed, and assay reaction conditions were optimized. The established assay was performed in a water bath for 50 minutes, and the amplification result was visualized directly as well as under ultraviolet (UV) light reflections. The detection limit of the RT-LAMP assay was 0.1pg total RNA of virus, which was one hundred-fold higher than that of RT-PCR. The results on specificity indicated that the assay had no cross-reactions with other subtype AIVs or avian respiratory pathogens. Furthermore, a total of 176 clinical samples collected from birds at the various live-bird markets (LBMs) were subjected to the H3-subtype-specific RT-LAMP (H3-RT-LAMP). Thirty-eight H3 subtype AIVs were identified from the 176 clinical samples that were consistent with that of virus isolation.

**Conclusions:**

The newly developed H3-RT-LAMP assay is simple, sensitive, rapid and can identify H3 subtype AIVs visually. Consequently, it will be a very useful screening assay for the surveillance of H3 subtype AIVs in underequipped laboratories as well as in field conditions.

## Background

Influenza A viruses are classified into subtypes consisting of16 hemagglutinin (HA) and 9 neuraminidase (NA) based on the antigenic differences of the HA and NA proteins, which are surface glycoproteins found on the viral envelope [1,3]. While the viruses with all subtypes can be detected in wild aquatic waterfowl, only few of the subtypes' influenza A viruses can infect mammalian species [1]. Avian influenza viruses (AIVs) can be divided into two distinct groups based on their virulence: the highly virulent viruses, including H5-and H7-subtype AIVs, are called highly pathogenic avian influenza (HPAI) and cause high mortality and morbidity; all other subtypes are categorized as low pathogenic avian influenza (LPAI), which cause mild or no symptoms in birds [2,4,5].

It has been shown that LPAIVs from birds can be a potential source of reassortant human influenza A viruses. In 1957, the virus that caused the influenza pandemic was found to posses three genes from subtype H2N2 from an avian virus and all remaining genes from a circulating human H1N1 virus [1]. Moreover, the Hong Kong influenza virus (H3N2) in 1968 was a reassortant with avian (H3) PB1 and HA genes and six other genes from human (H2N2) virus [6].

Currently, there is no evidence that H5N1 HPAIVs can transmit widely between humans; the human-pandemic influenza strains are all generated by gene reassortment between human influenza viruses, avian influenza viruses and swine influenza viruses [7]. The influenza A viruses spreading in humans recently include seasonal H3N2, H1N1 and pandemic influenza A H1N1 viruses. It's notable that the results of a recent epidemiological investigation into different HA subtype AIVs shows that the H3 subtype is the predominant subtype among LPAIVs, and the seasonal variations in isolation of H3 subtype AIVs are consistent with that of human H3 subtype influenza viruses [8]. Some research predicts that H3 subtype AIVs will get the ability of infecting human directly through gene reassortment [9,10]. Consequently, it is important to enhance surveillance for H3 subtype AIVs infections and to develop a simple, rapid and sensitive detection method.

Loop-mediated isothermal amplification (LAMP) is a new nucleic acid amplification method that uses a set of six primers that target the eight site of a conserved gene, yielding a large white pyrophosphate ion by-product. The result can be viewed by adding a fluorescent metal indicator to the reaction mix before amplification [11-13]. Another significant advantage is that the assay can be performed in a water bath within 30-60 minutes. Therefore, LAMP is simple, rapid and specific enough to detect pathogenic microorganisms in field conditions, and currently the assay is being used widely for the detection of various viral pathogens [14-22].

The purpose of the study reported here was specifically to enhance the surveillance of the H3 subtype of AIVs by developing and optimizing the LAMP technique for the rapid detection of H3 subtype AIVs in live birds.

## Methods

### Viral strains and DNA/RNA extraction

The reference strains of AIVs, clinical samples and other avian respiratory pathogens used in this study are listed in Table 1 and 2. The Genomic DNA/RNA was extracted from 200 ul samples using a DNA/RNA Miniprep Kit (Axygen Biosciences, Hangzhou, China), according to the protocol suggested by the manufacturer. The DNA and RNA were eluted with 40 ul elution buffer and were stored at -70°C immediately, until use.

**Table 1:** Clinic cloacal swab samples used to evaluate the feasibility of RT-LAMP assay.

Number	Sources and identification	Virus isolation	RT-PCR	RT-LAMP
1	A/Duck/Guangxi/N42/2009(H3N2)	+	+	+
2	A/Duck/Guangxi/M20/2009(H3N2)	+	+	+
3	A/Duck/Guangxi/04D3/2009(H3)	+	+	+
4	A/Duck/Guangxi/04D8/2009(H3)	+	+	+
5	A/Duck/Guangxi/017D5/2009(H3)	+	+	+
6	A/Duck/Guangxi/LZD11/2009(H3)	+	+	+
7	A/Duck/Guangxi/LZD15/2009(H3)	+	+	+
8	A/Duck/Guangxi/LZD23/2009(H3)	+	+	+
9	A/Duck/Guangxi/LZD27/2009(H3)	+	+	+
10	A/Duck/Guangxi/LZD28/2009(H3)	+	+	+
11	A/Duck/Guangxi/042D16/2009(H3)	+	+	+
12	A/Duck/Guangxi/046D6/2009(H3)	+	+	+
13	A/Duck/Guangxi/046D15/2009(H3)	+	+	+
14	A/Duck/Guangxi/048D617/2010(H3)	+	-	+
15	A/Duck/Guangxi/047D20/2010(H3)	+	+	+
16	A/Duck/Guangxi/047D15/2010(H3)	+	+	+
17	A/Duck/Guangxi/047D16/2010(H3)	+	+	+
18	A/Duck/Guangxi/049D7/2010(H3)	+	+	+
19	A/Duck/Guangxi/054D2/2010(H3)	+	+	+
20	A/Duck/Guangxi/054D3/2010(H3)	+	+	+
21	A/Duck/Guangxi/054D4/2010(H3)	+	+	+
22	A/Duck/Guangxi/054D7/2010(H3)	+	+	+
23	A/Duck/Guangxi/054D16/2010(H3)	+	+	+
24	A/Duck/Guangxi/054D19/2010(H3)	+	+	+
25	A/Duck/Guangxi/054D20/2010(H3)	+	+	+
26	A/Chicken/Guangxi/055C2/2010(H3)	+	+	+
27	A/Duck/Guangxi/057D19/2010(H3)	+	+	+
28	A/Duck/Guangxi/057D6/2010(H3)	+	+	+
29	A/Duck/Guangxi/WYD1/2010(H3)	+	+	+
30	A/Duck/Guangxi/066D16/2010(H3)	+	+	+
31	A/Duck/Guangxi/068D8/2010(H3)	+	+	+
32	A/Chicken/Guangxi/068C3/2010(H3)	+	+	+
33	A/Duck/Guangxi/070D8/2010(H3)	+	-	+
34	A/Duck/Guangxi/070D14/2010(H3)	+	-	+
35	A/Duck/Guangxi/072D5/2010(H3)	+	+	+
36	A/Duck/Guangxi/072D4/2010(H3)	+	+	+
37	A/Duck/Guangxi/072D2/2010(H3)	+	+	+
38	A/Duck/Guangxi/072D7/2010(H3)	+	+	+
39	A/Chicken/Guangxi/LF/2007	-	-	-
40	A/Duck/Guangxi/RX/2009	-	-	-
41	A/Chicken/Guangxi/015C7/2009	-	-	-
42	A/Duck/Guangxi/015D7/2009	-	-	-
43	A/Duck/Guangxi/011D3/2009	-	-	-
44	A/Duck/Guangxi/011D10/2009	-	-	-
45	A/Duck/Guangxi/016D10/2009	-	-	-
46	A/Duck/Guangxi/016D8/2009	-	-	-
47	A/Duck/Guangxi/022D1/2009	-	-	-
48	A/Chicken/Guangxi/037C10/2009	-	-	-
49	A/Duck/Guangxi/LAD9/2009	-	-	-
50	A/francolin/Nanning/018B-3/2010	-	-	-
51	A/francolin/Nanning/020B-7/2010	-	-	-
52	A/francolin/Nanning/022B12/2010	-	-	-
53	A/francolin/Nanning/022B13/2010	-	-	-
54	A/francolin/Nanning/022B7/2010	-	-	-
55	A/Chicken/Guangxi/DXC4/2010	-	-	-
56	A/Chicken/Guangxi/066C10/2010	-	-	-
57	A/Chicken/Guangxi/067C4/2010	-	-	-
58	A/Chicken/Guangxi/067C1/2010	-	-	-
59	A/Duck/Guangxi/068D18/2010	-	-	-
60	A/Duck/Guangxi/01D5/2009	-	-	-
61	A/Duck/Guangxi/02D6/2009	-	-	-
62	A/Duck/Guangxi/02D7/2009	-	-	-
63	A/Duck/Guangxi/04D5/2009	-	-	-
64	A/Duck/Guangxi/010D9/2009	-	-	-
65	A/Duck/Guangxi/011C5/2009	-	-	-
66	A/Duck/Guangxi/011C17/2009	-	-	-
67	A/Duck/Guangxi/011D5/2009	-	-	-
68	A/Duck/Guangxi/014D10/2009	-	-	-
69	A/Duck/Guangxi/014D15/2009	-	-	-
70	A/Duck/Guangxi/014D18/2009	-	-	-
71	A/Duck/Guangxi/016D7/2009	-	-	-
72	A/Duck/Guangxi/017C2/2009	-	-	-
73	A/Duck/Guangxi/017C12/2009	-	-	-
74	A/Duck/Guangxi/019C13/2009	-	-	-
75	A/Duck/Guangxi/026D10/2009	-	-	-
76	A/Duck/Guangxi/024C2/2009	-	-	-
77	A/Duck/Guangxi/024D2/2009	-	-	-
78	A/Duck/Guangxi/028D6/2009	-	-	-
79	A/Goose/Guangxi/027G10/2009	-	-	-
80	A/Duck/Guangxi/025D6/2009	-	-	-
81	A/Duck/Guangxi/026D21/2009	-	-	-
82	A/Duck/Guangxi/027D2/2009	-	-	-
83	A/Duck/Guangxi/033C1/2009	-	-	-
84	A/Duck/Guangxi/033C2/2009	-	-	-
85	A/Duck/Guangxi/033C3/2009	-	-	-
86	A/Duck/Guangxi/033C4/2009	-	-	-
87	A/Duck/Guangxi/033C5/2009	-	-	-
88	A/Duck/Guangxi/033C7/2009	-	-	-
89	A/Duck/Guangxi/033C9/2009	-	-	-
90	A/Duck/Guangxi/033C10/2009	-	-	-
91	A/Duck/Guangxi/031G2/2009	-	-	-
92	A/Duck/Guangxi/031G11/2009	-	-	-
93	A/Duck/Guangxi/031G12/2009	-	-	-
94	A/Duck/Guangxi/042D1/2009	-	-	-
95	A/Duck/Guangxi/042D3/2009	-	-	-
96	A/Duck/Guangxi/014D4/2009	-	-	-
97	A/Duck/Guangxi/042D9/2009	-	-	-
98	A/Duck/Guangxi/042D10/2009	-	-	-
99	A/Duck/Guangxi/042D19/2009	-	-	-
100	A/Duck/Guangxi/044D6/2009	-	-	-
101	A/Duck/Guangxi/044D7/2009	-	-	-
102	A/Duck/Guangxi/044D8/2009	-	-	-
103	A/Duck/Guangxi/044D10/2009	-	-	-
104	A/Duck/Guangxi/044D12/2009	-	-	-
105	A/Duck/Guangxi/044D17/2009	-	-	-
106	A/Duck/Guangxi/044C4/2009	-	-	-
107	A/Duck/Guangxi/044C7/2009	-	-	-
108	A/Duck/Guangxi/041D10/2009	-	-	-
109	A/Duck/Guangxi/041D11/2009	-	-	-
110	A/Duck/Guangxi/041D13/2009	-	-	-
111	A/Duck/Guangxi/048D15/2010	-	-	-
112	A/Duck/Guangxi/048D18/2010	-	-	-
113	A/Duck/Guangxi/048D2/2010	-	-	-
114	A/Duck/Guangxi/048D16/2010	-	-	-
115	A/Duck/Guangxi/PXD7/2010	-	-	-
116	A/Duck/Guangxi/PXD5/2010	-	-	-
117	A/Duck/Guangxi/PXD15/2010	-	-	-
118	A/Duck/Guangxi/052D16/2010	-	-	-
119	A/Duck/Guangxi/052D14/2010	-	-	-
120	A/Duck/Guangxi/052D8/2010	-	-	-
121	A/Duck/Guangxi/052D2/2010	-	-	-
122	A/Duck/Guangxi/052D1/2010	-	-	-
123	A/Duck/Guangxi/049D1/2010	-	-	-
124	A/Duck/Guangxi/049D2/2010	-	-	-
125	A/Duck/Guangxi/049D32010	-	-	-
126	A/Duck/Guangxi/049D4/2010	-	-	-
127	A/Duck/Guangxi/049D5/2010	-	-	-
128	A/Duck/Guangxi/049D8/2010	-	-	-
129	A/Duck/Guangxi/049D12/2010	-	-	-
130	A/Duck/Guangxi/049D15/2010	-	-	-
131	A/Duck/Guangxi/049D17/2010	-	-	-
132	A/Duck/Guangxi/049D18/2010	-	-	-
133	A/Duck/Guangxi/055D4/2010	-	-	-
134	A/Duck/Guangxi/055D5/2010	-	-	-
135	A/Duck/Guangxi/055D6/2010	-	-	-
136	A/Duck/Guangxi/055D7/2010	-	-	-
137	A/Duck/Guangxi/055D13/2010	-	-	-
138	A/Duck/Guangxi/055D20/2010	-	-	-
139	A/Duck/Guangxi/058D1/2010	-	-	-
140	A/Duck/Guangxi/058D6/2010	-	-	-
141	A/Duck/Guangxi/058D8/2010	-	-	-
142	A/Duck/Guangxi/058D12/2010	-	-	-
143	A/Duck/Guangxi/058D13/2010	-	-	-
144	A/Duck/Guangxi/058D15/2010	-	-	-
145	A/Duck/Guangxi/062D2/2010	-	-	-
146	A/Duck/Guangxi/062D3/2010	-	-	-
147	A/Duck/Guangxi/062D4/2010	-	-	-
148	A/Duck/Guangxi/062D5/2010	-	-	-
149	A/Duck/Guangxi/062D7/2010	-	-	-
150	A/Duck/Guangxi/062D8/2010	-	-	-
151	A/Duck/Guangxi/062D9/2010	-	-	-
152	A/Duck/Guangxi/062D10/2010	-	-	-
153	A/Duck/Guangxi/062D11/2010	-	-	-
154	A/Duck/Guangxi/062D12/2010	-	-	-
155	A/Duck/Guangxi/062D13/2010	-	-	-
156	A/Duck/Guangxi/062D14/2010	-	-	-
157	A/Duck/Guangxi/062D15/2010	-	-	-
158	A/Duck/Guangxi/062D17/2010	-	-	-
159	A/Duck/Guangxi/062D18/2010	-	-	-
160	A/Duck/Guangxi/062D19/2010	-	-	-
161	A/Duck/Guangxi/066D2/2010	-	-	-
162	A/Duck/Guangxi/066D5/2010	-	-	-
163	A/Duck/Guangxi/066D7/2010	-	-	-
164	A/Duck/Guangxi/066D10/2010	-	-	-
165	A/Duck/Guangxi/066D20/2010	-	-	-
166	A/Duck/Guangxi/BSD20/2010	-	-	-
167	A/Duck/Guangxi/BSD12/2010	-	-	-
168	A/Duck/Guangxi/BSD18/2010	-	-	-
169	A/Duck/Guangxi/BSD23/2010	-	-	-
170	A/Duck/Guangxi/BSD38/2010	-	-	-
171	A/Duck/Guangxi/070D5/2010	-	-	-
172	A/Duck/Guangxi/072D14/2010	-	-	-
173	A/Duck/Guangxi/072D16/2010	-	-	-
174	A/Duck/Guangxi/072D13/2010	-	-	-
175	A/Duck/Guangxi/072D19/2010	-	-	-
176	A/Duck/Guangxi/072D1/2010	-	-	-

**Table 2:** Virus strains used for specificity of H3-RT-LAMP assay

Number	Virus strain	H3-RT-LAMP
1	A/Duck/Guangxi/030D/2009(H1N1)	**-**
2	A/Mallard/Alberta/77(H2N3)	**-**
3	A/Mallard/Alberta/85(H3N6)	**+**
4	A/Duck/Guangxi/N42/2009(H3N2)	**+**
5	A/Duck/Guangxi/027D/2009 (H4)	**-**
6	A/Turkey/GA/209092/02(H5N2)	**-**
7	A/Turkey/CA/35621/84(H5N3)	**-**
8	A/waterfowl/GA/269452-56/03(H5N7)	**-**
9	A/Turkey/MA/40550/87-Bel42(H5N1)	**-**
10	A/Turkey/WI/68(H5N9)	**-**
11	A/Turkey/Ontario/63(H6N8)	**-**
12	A/Chicken/NY/273874/03(H7N2)	**-**
13	A/Turkey/Ontario/6118/67(H8N4)	**-**
14	A/Duck/Guangxi/RX/09(H9N2)	**-**
15	A/Turkey/MN/24838-590/79(H10N7)	**-**
16	A/Chicken/Guangxi/43C/09(H11)	**-**
17	A/Duck/Alberta/60/76(H12N5)	**-**
18	A/Gull/MD/704/77(H13N6)	**-**
19	A/Duck/Australia/341/83(H15N8)	**-**
20	Newcastle disease virus (Lasota)	**-**
21	Infectious bronchitis virus (M41)	**-**
22	Infectious Laryngotracheitis virus	**-**
23	*Mycoplasma gallisepticum *(S6)	**-**

### Design of primers for the RT-LAMP assay

**Figure 1:**
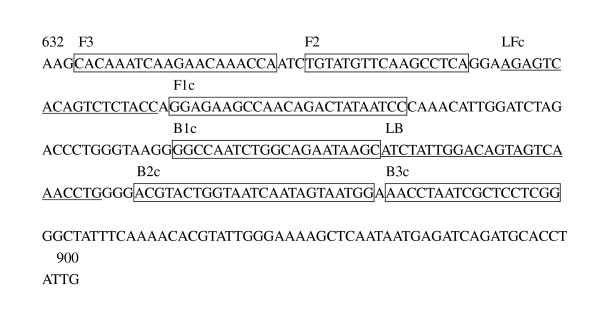
**Positions of RT-LAMP primers on HA gene of H3 subtype AIVs**. Locations of primers binding sequences are underlined and boxed. The referenced sequence A/Duck/GuangXi/N42/2009(H3N2) can be obtained in GenBank (accession number JN003630).

According to the sequences of the H3 subtype AIVs' hemagglutinin (HA) gene available in GenBank (accession no. JN003630) and the sequences of viruses isolated in China, several primer sets of RT-LAMP assays were designed using the LAMP primer design software Primer Explorer V4 http://primerexplorer.jp/elamp4.0.0/index.html. Finally, an optimal set was chosen after many comparable experiments to ensure the highest sensitivity and specificity of the RT-LAMP assay. The RT-LAMP primer set comprising two outer primers (forward primer F3 and backward primer B3), two inner primers (forward inner primer FIP and backward inner primer BIP), and two loop primers (forward loop primer LF and backward loop primer LB) recognized eight sites on the target sequence specific to the HA gene. The details for the primers are shown in Table 3 and Figure 1.

**Table 3:** Sequences of primers designed for RT-LAMP assay

Primer name	Sequence(5'-3')^a^	Genome position
FIP	GGATTATAGTCTGTTGGCTTCTCC-GAATTC-TGTATGTTCAAGCCTCA	658-720
BIP	GGCCAATCTGGCAGAATAAGC-GATATC-CCATTACTATTGATTACCAGTACGT	750-823
F3	CACAAATCAAGAACAAACCA	635-654
B3	CCGAGGAGCGATTAGGTT	825-842
LF	GGTAGAGACTGTGACTCT	678-695
LB	ATCTATTGGACAGTAGTCAAACCTG	771-795

^a ^The restriction enzyme site sequences of *EcoR I *and *EcoR V *were added in FIP and BIP primer respectively. Primers of F3, B3 were applied to the RT-PCR assay of H3 subtype AIVs.

### Optimization of the RT-LAMP conditions

The RT-LAMP assay was carried out in a conventional water bath with 25 μl of reaction mixture (Table 4). The concentration of reactions component comprised primers (synthesized by Invitrogen), MgSO_4 _(Sigma), Betaine (Sigma), deoxynucleoside triphosphate (dNTP), RNA template, RevertAid™ M-MuLV Reverse Transcriptase (MBI Fermentas) and Bst DNA polymerase (New England Biolabs) were optimized. To visualize the reaction, 25 μmol/L Calcein (International Laboratory, USA) and 0.5 mmol/L MnCl_2 _(International Laboratory, USA) had been added previously to the reaction mixture, as suggested by the related reports [13]. The amplification reaction was performed in a water bath at 59°C, 60°C, 61°C, 62°C, 63°C, 64°C, and 65°C for 30, 45, and 60 min respectively to find the optimal temperature and time. Then the reaction was terminated by heating at 80°C for 5 min. All of the experiments were repeated three times.

**Table 4:** The reaction system of RT-LAMP assay

Reaction component		Reaction volume of H3-RT-LAMP assay
10 × Thermopol Buffer		2.5 μL
MgSO_4_		5 mmol/L
Betaine		1 mmol/L
	F3	0.2 μmol/L
	B3	0.2 μmol/L
primer	FIP	1.6 μmol/L
	BIP	1.6 μmol/L
	LF	0.8 μmol/L
	LB	0.8 μmol/L
dNTP		1.4 mmol/L
Calcein		25 μmol/L
MnCl_2_		0.5 mmol/L
M-MuLV RTase		200U
Bst DNA polymerase		8U
Template RNA		2 μL
Nuclease-free water		Up to 25 μL

### RT-PCR

Prior to this study, there was no official primer or procedure for RT-PCR to detect H3 subtyping of AIVs. The outer primers of RT-LAMP (forward primer F3 and backward primer B3) that were specific to target sequence and had high priming efficiency were applied in RT-PCR assay of H3 subtype AIVs. The reaction condition of RT-PCR was optimized to get the highest sensitivity of amplification. Finally, the RT-PCR assay was carried out in a 50 ul reaction volume containing 10 mM of each dNTP, 5 ul of 5 × RT Reaction Buffer, 200 U of RevertAid™ M-MuLV Reverse Transcriptase, 5 ul of 10 × Taq Buffer, 5 U of Taq DNA polymerase (MBI Fermentas), 10 uM each of B3 and F3 primers (Table 3), and 1ul of extracted RNA, DEPC-treated water up to 50 ul.

The reaction was performed in a Thermal Cycler (BIO-RAD) at 42°C for 60 min and at 94°C for 5 min; followed by 30 cycles of 94°C for 40 s, 55°C for 30 s, and 72°C for 30 s; and a final extension for 10 min at 72°C. Then the amplified products were analyzed using 1% agarose gel electrophoresis.

### Analysis of RT-LAMP assay products

For the visual inspection of the products of the RT-LAMP assay, white magnesium pyrophosphate precipitations in the tube were centrifuged at 8,000 X g for 5 min in a Biofuge centrifuge (Primo R, HERAEUS). Meanwhile, color changes of the reaction mixture were observed directly under daylight and UV light.

### Agarose gel electrophoresis analysis and DNA sequencing

Five μl of the RT-LAMP assay product was analyzed using 1.5% agarose gel electrophoresis. To test the specificity of the RT-LAMP assay product, the RT-LAMP amplicons were first digested with *EcoR*I and *EcoR*V restriction enzymes using the suggested protocol and then analyzed with 1.5% agarose gel electrophoresis. The products digested by restriction enzymes were purified using an AxyPrep ™ DNA Gel Extraction Kit and were sequenced by Invitrogen Company.

### Sensitivity and specificity of RT-LAMP assay

The detection limit of RT-LAMP was determined by testing serial 10-fold dilutions of total RNA of A/Duck/Guangxi/N42/2009 (H3N2) respectively, and the same concentration samples were comparatively detected by RT-PCR. All of the experiments were repeated three times. The concentration range of total RNA in the diluted sample was 1ng/tube to 1fg/tube, measured by ultraviolet spectrophotometer (Beckman UV-800).

To evaluate the specificity of the primer set used for the RT-LAMP assay, DNA/RNA extracted from different subtype AIVs and from Newcastle disease virus (NDV), infectious bronchitis virus (IBV), infectious laryngotracheitis virus (ILTV), *Mycoplasma gallisepticum *(MG) were detected by RT-LAMP assay. The amplification results were analyzed using 1.5% agarose gel electrophoresis and the color changes of reaction mixture were inspected under daylight and UV light.

### Detection of clinical specimens by RT-LAMP assay

A total of 176 cloacal swabs were collected from poultry at LBMs. The clinical specimens were prepared in a viral transport medium, which was made up of 0.05 M phosphate buffered saline (PBS) containing antibiotics of penicillin (10000 units/ml), streptomycin (10 mg/ml), gentamycin (10 mg/ml), kanamycin (10 mg/ml) and 5% (v/v) fetal bovine serum. Cloacal swabs samples were injected into 9-to 11-day-old embryonated specific-pathogen-free (SPF) chicken eggs as previously described [5]. Allantoid fluids were recovered 48-72 hrs after incubation for virus detection and titration as described [23]. All clinical specimens were tested by RT-LAMP, RT-PCR, and virus isolation, respectively.

## Results

### The optimal protocol of RT-LAMP assay and inspection of products

The RT-LAMP reaction was optimized in a 25 ul total reaction mixture and performed in a conventional water-bath for 45 min at 63°C and then for 5 min at 80°C to terminate the reaction. The details of optimize reaction solution of RT-LAMP assay are shown in Table 4.

**Figure 2:**
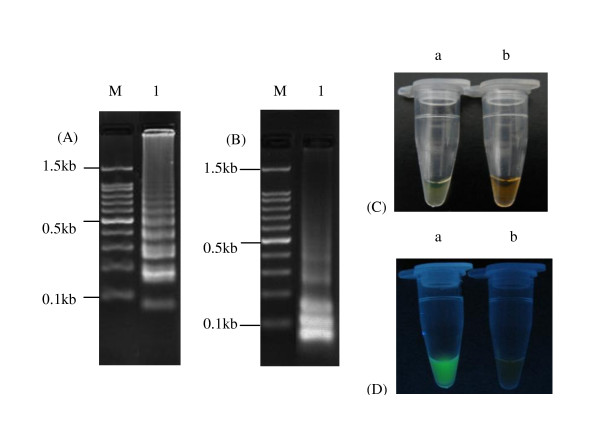
**Detection of H3 subtype AIVs by RT-LAMP assay**. (A) Products of RT-LAMP assay confirmed by 1.5% agarose gel electrophoresis. (B) Products of RT-LAMP assay digested by *EcoR *I and *EcoR *V restriction enzyme. (C) Result of RT-LAMP assay under daylight. (D) Result of RT-LAMP assay under UV light. M:100 bp. a: positive sample. b: negative sample.

The positive results of RT-LAMP assay showed typical ladder pattern by 1.5% agarose gel electrophoresis (Figure 2-A). Furthermore, a large amount of DNA was synthesized, yielding a large white magnesium pyrophosphate by-product, so the white magnesium pyrophosphate on the bottom of the tube could be inspected after centrifugation. Alternatively, a color change in the reaction solution could be seen with the naked eye: the solution changed from orange to green for a positive reaction and remained orange for the negative reaction (Figure 2-C). In addition, the positive sample showed a strong green fluorescence under UV light (Figure 2-D). The products of the RT-LAMP assay digested by *EcoR *I and *EcoR *V restriction enzymes displayed predicted fragments (Figure 2-B). These fragments were purified and then confirmed by sequencing analysis (date not shown).

### Specificity and sensitivity of RT-LAMP assay for detection of H3 Subtype AIVs

**Figure 3:**
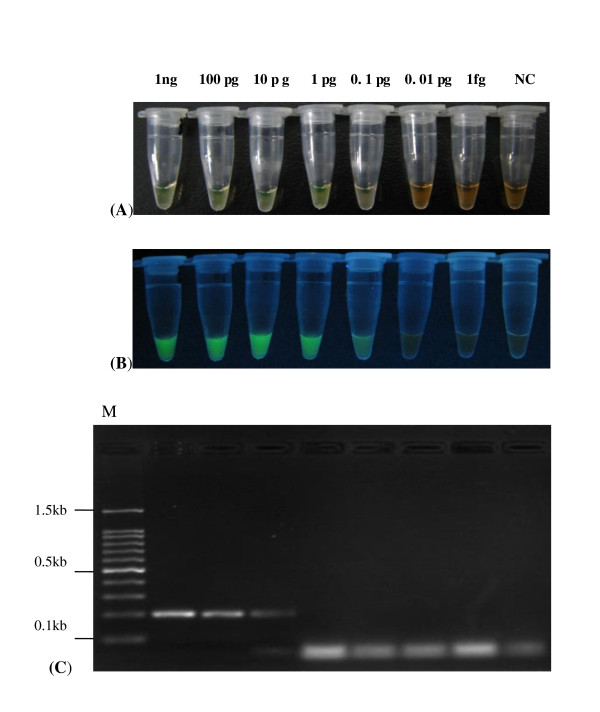
**Comparative sensitivity tests between RT-LAMP and RT-PCR assays for detection of H3 subtype AIVs (the detection range of virus total RNA was 1ng/tube to 1fg/tube)**. (A) The result of sensitivity test for RT-LAMP assay under daylight. (B) The result of sensitivity test for RT-LAMP assay under UV light. (C) The result of sensitivity test for RT-PCR. M: 100 bp. NC: negative control.

The viral DNA/RNA extracted from the different subtype AIVs and NDV, IBV, ILTV, MG were detected using the RT-LAMP assay to evaluate the specificity. The results of the negative sample showed no typical ladder pattern by 1.5% agarose gel electrophoresis and also had no color change detectable under daylight or UV light, which indicated that RT-LAMP assay had no cross reaction with other viruses (Table 2). The detection limit of RT-LAMP assay was 0.1 pg total RNA of virus, which was 100-fold higher than that of RT-PCR. The results of the visual detection of sensitivity tests are shown in Figure (3A, B and 3C).

### Evaluation of RT-LAMP assay with clinical Samples

A total of 176 random cloacal swab samples were collected from poultry at various LBMs and were tested by RT-LAMP, RT-PCR, and virus isolation respectively (Table 1). The results of virus isolation showed that there were 38 positive samples of H3 subtype AIVs among the 176 cloacal swab samples, which was consistent with that of RT-LAMP assay; however, three positive samples were missed by RT-PCR. The results of statistical analysis showed that comparative detection of H3 subtype AIVs from cloacal swab samples by RT-LAMP was not statistically significant from RT-PCR and virus isolation (Table 5).

**Table 5:** Comparative detection of cloacal swab samples by virus isolation, RT-PCR and RT-LAMP

Results	**Virus isolation**^**a**^	**RT-PCR**^**b**^	**RT-LAMP**^**c**^
Positive	38	35	38
Negative	138	141	138
Total	176	176	176
Sig.(P value)	a-b:0.696	b-a:0.696	c-a:1.000
	a-c:1.000	b-c:0.696	c-b:0.696

^a^Virus isolation, ^b^RT-PCR, ^c^RT-LAMP.

The significant differences among the three detection methods were analyzed by SPSS software. The default significance level is 0.05.

## Discussion

The H3 subtype AIVs can provide genes for human influenza virus through gene reassortment, which raises great concerns in terms of its potential threat to human health [6]. Consequently, the development of a rapid, simple, sensitive detection method for H3 subtype AIVs is required. So far, there are several PCR-based methods being used to detect AIVs, but they all need precision instruments to amplify the nucleic acid of target sample; therefore, they can't be applied in field conditions [24,25]. The loop-mediated isothermal amplification (LAMP) assay is a nucleic acid amplification method developed by Notomi et al. that does not require any specialized equipment [11] and can be performed in a water bath or a heating block at an isothermal temperature between 60°C to 65°C within 30 to 60 min. In contrast, the conventional PCR method takes at least one hour.

The LAMP assay relies on autocycling strand displacement DNA synthesis performed by Bst DNA polymerase with a high degree of strand displacement activity, which leads to an excellent sensitivity. The primer set of LAMP assay comprise two outer primers (forward primer F3 and backward primer B3), two inner primers (forward inner primer FIP and backward inner primer BIP), and two loop primers (forward loop primer LF and backward loop primer LB) and it recognizes eight sites on the target sequence specific to HA gene, so it's specificity is very high. The viral RNA can be detected by the one-step RT-LAMP assay. When this is performed with reverse transcriptase and LAMP reaction mixture, the reverse transcription and DNA amplification can be accomplished at a constant temperature within 60 min.

During amplification, a large amount of white magnesium pyrophosphate is produced, which can be inspected directly with the naked eye. Alternatively, the result of the RT-LAMP assay can be visualized by adding fluorescence reagent (Calcein and MnCl_2_) into the reaction mixture to observe the change of the reaction color under day light or UV light [11-13]. Before the amplification reaction, calcein is combined with manganese ion to get the quenching effect, so the reaction solution is orange. Along with LAMP reaction processing, pyrophosphate ions remove manganese ions from calcein, resulting in greater fluorescence, which indicates the presence of the target gene [12].

In the present study, we first developed a rapid and sensitive RT-LAMP assay to visually detect H3 subtype AIVs. According to the sequences of the HA gene of H3 subtype AIVs available in GenBank and the sequences of H3 subtype AIVs isolated in China, we designed several sets of primers and finally chose the optimal set after many comparative experiments to ensure the high specificity and sensitivity of the RT-LAMP assay.

The proportions of the reagents in the reaction mixture were optimized to develop a stable RT-LAMP assay with high sensitivity. The detection limit of the RT-LAMP assay was 0.1 pg total RNA of virus, which was 100-fold higher than that of RT-PCR. The results of the specificity test showed that the assay had no cross reaction with other subtype AIVs and avian respiratory pathogens. Furthermore, a total of 176 cloacal swab samples collected from LBMs were tested using RT-LAMP, RT-PCR, and virus isolation respectively. The results showed that the clinical sensitivity of the RT-LAMP assay was consistent with virus isolation.

Although the RT-LAMP assay has many advantages over the similar nucleic acid amplification method, there is still a problem to note. Because of the high sensitivity of the RT-LAMP assay, a micro amount of RNA contamination in reagents, environment and instruments, such as the pipette, can result in a false positive. Moreover, the RT-LAMP assay has great amplification efficiency so the reaction product can form aerosol to contaminate the surroundings when opening the tube. To avoid the contamination in this study, the fluorescence reagent (Calcein and MnCl_2_) were added to RT-LAMP reaction mixture before amplification in order to inspect the result of RT-LAMP assay directly with the naked eye.

## Conclusions

In this study, the established RT-LAMP assay with high sensitivity was performed in a water bath within only 50 min, and the amplification results were visualized by adding fluorescence reagent. In summary, the newly developed assay can be used as one important method for detecting H3 subtype AIVs in field conditions with no need for specialized equipment.

## Competing interests

The authors declare that they have no competing interests.

## Authors' contributions

YP, ZXX and MIK designed the experiments. QF and LJX prepared the RNA samples. YP designed the primers and optimized conditions of the RT-LAMP assay. YP, JBL, YSP, XWD and ZQX carried out the experiments shown in Figure 2 and 3 and in Table 1, 4, 5. JXF performed the data analysis. YP and MIK wrote the manuscript. All authors read and approved the final manuscript.
